# The Antihypertensive Drug Nifedipine Modulates the Metabolism of Chondrocytes and Human Bone Marrow-Derived Mesenchymal Stem Cells

**DOI:** 10.3389/fendo.2019.00756

**Published:** 2019-11-08

**Authors:** Ilona Uzieliene, Eiva Bernotiene, Greta Rakauskiene, Jaroslav Denkovskij, Edvardas Bagdonas, Zygmunt Mackiewicz, Narunas Porvaneckas, Giedrius Kvederas, Ali Mobasheri

**Affiliations:** ^1^Department of Regenerative Medicine, State Research Institute Centre for Innovative Medicine, Vilnius, Lithuania; ^2^Faculty of Medicine, Vilnius University, Vilnius, Lithuania; ^3^Research Unit of Medical Imaging, Physics and Technology, Faculty of Medicine, University of Oulu, Oulu, Finland; ^4^Centre for Sport, Exercise and Osteoarthritis Research Versus Arthritis, Queen's Medical Centre, Nottingham, United Kingdom; ^5^Sheik Salem Bin Mahfouz Scientific Chair for Treatment of Osteoarthritis With Stem Cells, King Abdulaziz University, Jeddah, Saudi Arabia

**Keywords:** chondrocytes, BMMSCs, nifedipine, BayK8644, energy metabolism, calcium channels

## Abstract

Aging is associated with the development of various chronic diseases, in which both cardiovascular disorders and osteoarthritis are dominant. Currently, there is no effective treatment for osteoarthritis, whereas hypertension is often treated with L-type voltage-operated calcium channel blocking drugs, nifedipine being among the most classical ones. Although nifedipine together with other L-type voltage-operated calcium channel inhibitors plays an important role in controlling hypertension, there are unresolved questions concerning its possible effect on cartilage tissue homeostasis and the development of osteoarthritis. The aim of this study was to analyse the effects of nifedipine on metabolic processes in human chondrocytes and bone marrow mesenchymal stem cells. To better understand whether the metabolic effects are mediated specifically through L-type voltage-operated calcium channel, effects of the agonist BayK8644 were analyzed in parallel. Nifedipine downregulated and mitochondrial respiration and ATP production in both cell types. Analysis of cartilage explants by electron microscopy also suggested that a small number of chondrocyte mitochondria's lose their activity in response to nifedipine. Conversely, nifedipine enhanced glycolytic capacity in chondrocytes, suggesting that these cells have the capacity to switch from oxidative phosphorylation to glycolysis and alter their metabolic activity in response to L-type voltage-operated calcium channel inhibition. Such a metabolic switch was not observed in bone marrow mesenchymal stem cells. Nitric oxide activity was upregulated by nifedipine in bone marrow mesenchymal stem cells and particularly in chondrocytes, implying its involvement in the effects of nifedipine on metabolism in both tested cell types. Furthermore, stimulation with nifedipine resulted in elevated production of collagen type II and glycosaminoglycans in micromass cultures under chondrogenic conditions. Taken together, we conclude that the antihypertensive drug nifedipine inhibits mitochondrial respiration in both chondrocytes and bone marrow mesenchymal stem cells and that these effects may be associated with the increased nitric oxide accumulation and pro-inflammatory activity. Nifedipine had positive effects on the production of collagen type II and proteoglycans in both cell types, implying potentially beneficial anabolic responses in articular cartilage. These results highlight a potential link between antihypertensive drugs and cartilage health.

## Introduction

Cardiovascular diseases (CVD) are important co-morbidities in many osteoarthritis (OA) patients and there is emerging evidence that they are mechanistically linked to the progression of OA (1–3). Arrhythmia, hypertension and cardiac ischemia are most prevalent in elderly and obese individuals, with limited physical activity and in many cases, with hormonal imbalance and metabolic disorders (4, 5).

The pathogenesis of OA and cardiovascular disorders might involve a common but neglected link, and altered intracellular calcium (iCa^2+^) signaling, which may contribute to the development of both pathologies in the same individual (6). Furthermore, modulators of ion channels, used for the treatment of cardiovascular diseases, particularly 1,4-Dihydropyridines (1,4-DHP), may also affect the structure and function of articular cartilage. Nifedipine (chemical name: 3,5-dimethyl 2,6 -dimethyl-4-(2-nitrophenyl)-1,4-dihydropyridine-3,5-dicarboxylate; marketed under the brand name Adalat among others) is one of the most commonly prescribed 1,4-DHP derived drugs for hypertension. It specifically blocks L-type voltage-operated calcium channels (VOCCs) in the vascular system and therefore, lowers high blood pressure (7–9). However, the effects of ion channel modulators on chondrocyte function and the progression of OA are not fully understood and need to be elucidated. Several studies suggest that calcium channel antagonists (especially L-type Ca^2+^ channel inhibitors) may have a beneficial effect on OA by attenuating its progression (10, 11). In this case, blockage of those channels by cardiovascular drugs may normalize heart rate and blood pressure as well as attenuate OA development and shift toward chondroprotection. However, there is insufficient mechanistic information on how L-type Ca^2+^ channel inhibition may potentially affect chondrocyte and cartilage function.

Recently, it has been shown that various 1,4-DHP calcium antagonists stimulate nitric oxide (NO) release (12–14). The majority of described NO effects are related to pro-inflammatory processes or mitochondrial dysfunction. The role of NO in articular cartilage damage was widely reviewed by Lotz (15). Among the effects discussed, there are inhibition of collagen and proteoglycan synthesis, induction of chondrocyte apoptosis, stimulation of metalloproteinase production and activation. Thus, NO appears to be a potential downstream mediator of nifedipine activity or at least contributes to the above-mentioned indirect effects.

Since both hypertension and OA sometimes coincide in same patients, the use of antihypertensive drugs may have effects on the metabolism of articular cartilage. The altered metabolic pathways in OA cartilage have been highlighted as potential therapeutic targets (16), therefore, the potential impact of antihypertensive drugs on cartilage metabolism needs careful attention. We have raised a novel hypothesis that VOCC inhibitors, used to treat hypertension, may simultaneously modulate iCa^2+^ levels in chondrocytes, leading to the functional, metabolic, and inflammatory responses, which in turn affect physiology of articular cartilage and production of extracellular matrix. Bone marrow mesenchymal stem cells (BMMSCs) are considered potential contributors to cartilage repair and regeneration due to their ability to undergo chondrogenesis upon exposure to specific factors (17, 18). Therefore, in the present study, the effects of nifedipine on BMMSCs and chondrocytes were investigated and compared. The aim of this study was to analyse metabolic and functional responses, including proliferation, mitochondrial respiration, glycolysis, NO activity, and extracellular matrix production in chondrocytes and BMMSCs, stimulated with nifedipine. These data could broaden our understanding on the effects of VOCC inhibitors used for treatment of hypertension and give some mechanistic insight on their role in development and progression of OA, potentially offering new targets for cartilage protection and promotion of regeneration.

## Materials and Methods

### Cell Isolation and Culture

Human articular cartilage samples were obtained from Vilnius University Hospital Santaros Klinikos as post-operative tissues during articular surgery from patients with OA (*n* = 5) (60–80 years age). Cartilage was dissected from anatomical locations with morphologically similar lesions. The excised pieces of cartilage were further chopped into small pieces, and incubated overnight in low glucose (1 g/L) Dulbecco's modified Eagle's medium (DMEM) medium (Merck Millipore, Darmstadt, Germany) without FBS at 37°C and 5% CO_2_. The next day, minced cartilage was washed with phosphate buffered saline (PBS) and incubated 1 h in pronase solution (26.5 U/mL) (Roche diagnostics, Indianapolis, Indiana, USA) at 37°C and 5% CO_2_ under conditions of constant shaking. Then, cartilage explants were washed twice with PBS, chopped into smaller pieces and transferred into a new 50 mL tube for the following chondrocytes isolation with type II collagenase. Ten microliter of type II collagenase solution (545 U/mL) (Biochrom AG, Berlin, Germany) were prepared per 1 g of cartilage sample cartilage sample. Cartilage pieces were incubated at 37°C and 5% CO_2_ for 3–4 h under conditions of constant shaking. After incubation, the digested solution was filtered through cell strainers of 100 and 70 μm. The enzymatic activity of collagenase was stopped by adding a double volume of complete medium—DMEM (1 g/L glucose), supplemented with 10% FBS (Merck Millipore, Darmstadt, Germany), 1% penicillin/streptomycin (Gibco, Life Technologies, Waltham, Massachusetts, USA). Cell filtrate was centrifuged for 5 min at 400 g, supernatant discarded and cell pellet resuspended in complete medium. Collected chondrocytes were expanded in tissue culture flasks (Gibco, Life Technologies, Waltham, Massachusetts, USA) with complete medium, and cultured in 37°C incubator with 5% CO_2_. The medium was changed twice a week. After reaching the confluence (~80%) cells were detached using trypsin-EDTA 0.25% solution (Gibco, Life Technologies, Waltham, Massachusetts, USA), counted (CASY, Omni Life Science, Bremen, Germany) and sub-cultured. Human BMMSCs were isolated from bone marrow tissues remaining after post-trauma surgical procedures from five healthy donors (50–60 years age), according to the established protocols by Center for Innovative Medicine (IMC). BMMSCs were characterized by typical MSC surface marker expression—CD44, CD73, CD90, CD105, and lack of hematopoietic surface marker expression CD14, CD34, CD45 as well as the ability to differentiate toward adipogenic, osteogenic, and chondrogenic lineages. BMMSCs were cultured under the same conditions as chondrocytes—in complete DMEM medium, but with addition of 1 ng/mL fibroblast growth factor 2 (FGF2)(Thermo Fisher Scientific, Vilnius, Lithuania). All the procedures made with human tissues within this study were approved by Bioethics Committee, permission No. 158200-14-741. All experiments were performed using chondrocytes and BMMSCs at passages (P) P2 to P3.

### Transmission Electron Microscopy Study of Cartilage Explants

Samples of cartilage tissue were dissected from the locations with morphologically similar lesions. Biopsy needles (3 mm, Integra Miltex, Vienna, Austria) were used to extract explant. Explants were weighed and put into 6-well plate, 100 mg of explants/well. Explants were separated into 2 groups—control group (chondrogenic medium only) and nifedipine exposure group [chondrogenic medium and nifedipine (10 μM) (Sigma-Aldrich, Hamburg, Germany)], and cultured for 7 days. The medium was changed at day 3 and 5. Nifedipine (10 μM) was added accordingly with each medium change. After 7 days explants were prepared for electron microscopy analysis. For transmission electron microscopy analysis the *ex vivo* cultivated cartilage explants derived from smooth either eroded articular surface were fixed in 2% glutaraldehyde in 0.1 M cacodylate buffer for 3 h, then with 1% OsO_4_ in the same buffer for 1 h, alcohol dehydrated, and embedded in araldite. Ultrathin sections were prepared on a Leica EM UC6 ultratome and stained with uranyl acetate and lead citrate. The observations of ultrathin sections were carried out with Philips/FEI (Morgagni) transmission electron microscope at magnification 3000x−10000x, and photos made using the Gatan digital camera.

### Proliferation Assay

Chondrocytes and BMMSCs were seeded into 12-well plates (SPL, Life Sciences, Gyeonggi-Do: South Korea) at a density of 20,000 cells/well in a complete medium. The next day, cells were divided into three treatment groups: control (with dimethyl sulfoxide (DMSO), which is a solvent for nifedipine and BayK8644 and was added the same amount as nifedipine and BayK8644 were), nifedipine (10 μM) and BayK8644 (10 μM) (Sigma-Aldrich, Hamburg, Germany). Cell proliferation was determined at days 1, 3, 5, 8, and 12 with cell counting kit -8 (CCK-8) (Dojindo, Munich, Germany) according to the manufacturer's instructions. Commercial CCK-8 kit allows to measure cell proliferation and cytotoxicity at once, by utilizing highly water-soluble tetrazolium salt. This salt is being reduced by living cell dehydrogenases and produces soluble orange formazan dye. This way the amount of the formazan dye generated by dehydrogenases in cells is directly proportional to the number of living cells. The medium was collected to 96 well plate (Orange Scientific, Braine-l'Alleud, Belgium) and absorbance at 450 nm was quantified with SpectraMaxvi3 spectrophotometer (Molecular Devices, San Jose, California, USA).

### Metabolic Analysis

Cellular metabolism was measured using Agilent Seahorse xFe24 metabolism analyzer and Mito-stress test kit (Agilent Seahorse, Santa Clara, California, USA). Agilent Seahorse metabolism analyser provides an informative study of cells energy metabolism. The sequential compound injection allows to measure basal cell respiration capacity, ATP production, maximal respiration rate and spare respiration capacity during mitochondrial respiration, and glycolysis, glycolytic capacity and glycolytic reserve during glycolysis.

The cells were seeded into Seahorse 24-well plates at a density 30,000 cells/well. The next day cells were divided into treatment groups: (1) control, which was cultivated in complete medium; (2) instant nifedipine, where nifedipine (10 μM) was added only during measurement; (3) nifedipine (10 μM) and BayK8644 (10 μM) for long term (24 h) incubation, and (4) nifedipine (10 μM) for 24 h and additional nifedipine (10 μM) added during measurement, for long and instant effect of the drug. Each group was measured in triplicates. After the treatment, complete cell medium was switched to Seahorse XF base medium (Agilent Technologies) supplemented with 10 mM glucose, 2 mM GlutaMAX (Gibco, Waltham, Massachusetts, USA) and 1 mM sodium pyruvate, and further incubated in CO_2_-free incubator for 1 h. The measurement was completed according to Agilent recommendations, by adding oligomycin, FCCP (carbonyl cyanide p trifluoromethoxyphenylhydrazone) and antimycin A/rotenone, which were prepared in Seahorse XF assay medium (Agilent Technologies) with a final concentration of 1, 0,5, and 1 μM. For glycolysis test, Glucose stress-test kit was used (Agilent Technologies). The cells were prepared the same way, only using Seahorse medium supplemented with 1 mM GlutaMAX (Gibco, Life Technologies, Waltham, Massachusetts, USA). During measurement, glucose, oligomycin and 2-deoxy-glucose (2-DG) were added with a final concentration of 100, 10, and 500 mM, respectively. After the measurement, the protein analysis was performed using Lowry method and the results were normalized to the amount of protein.

### Intracellular Calcium Study

For intracellular calcium studies chondrocytes and BMMSCs were seeded into 12-well plates (SPL, Life Sciences, Gyeonggi-Do: South Korea) at density 50,000 cells/well in a complete medium. After the cells reached confluence, the wells were subdivided into 6 different groups (2 for each treatment) and treated for 24 h with: (1) DMSO, (2) 10 μM of nifedipine, (3) 10 μM of BayK8644, (4) 1 ng/mL IL-1β (Prospec, Ness-Ziona, Israel), (5) 1 ng/mL IL-1β + 10 μM nifedipine, (6) 1 ng/mL IL-1β + 10 μM BayK8644. At the end cells were detached and stained with 1 μM of calcium specific fluorescent dye Cal-520 (Interchim, Montlucon, France) for 30 min, 37°C, then measured using flow cytometer (Calibur) and the data were analyzed using FlowJo (FlowJo, USA) software.

### Nitric Oxide Accumulation Study

The intracellular nitric oxide activity was assessed in chondrocytes and BMMSCs, treated for 24 h with: (1) 10 μM of DMSO (control), (2) 10 μM of nifedipine, (3) 10 μM of BayK8644 and (5) 1 μl/mL of cigarette smoke extract (CSE) (Murty Pharmaceuticals, Lexington, USA), as a positive control. The next day cells were detached and measured for nitric oxide activity by using MUSE flow cytometer (Merck Millipore, Darmstadt, Germany) and Muse™ Nitric Oxide Kit (Merck, Germany), which includes nitric oxide reagent and the dead cell dye 7-AAD, according to manufacturer's instruction.

### Chondrogenic Differentiation Study

Chondrogenesis was induced in chondrocytes and BMMSCs using standard protocol used by State Research Institute Center for Innovative medicine. Chondrogenic medium included high glucose (4.5 g/L) DMEM medium (Merck Millipore), 1% penicillin/streptomycin, 1% insulin-transferrin-selenium (all from Gibco Life Technologies), L-proline (350 nM) (Carl Roth, Karlsruhe, Germany), dexamethasone (100 nM) (Sigma Aldrich), ascorbic acid-phosphate (170 nM) (Sigma Aldrich) and TGF-β3 (10 ng/mL) (Gibco, Life Technologies). Incomplete chondrogenic medium (the same constituents without TGF-β3) was used as control.

Furthermore, control and TGF-β3 groups were subdivided into 3 subgroups: (1) with addition of DMSO, which is a solvent for nifedipine and BayK8644, (2) with nifedipine (10 μM), and (3) BayK866 (10 μM). In total, 6 subgroups of different stimulation conditions were applied for cell cultivation in pellets in 15 mL tubes (Gibco, Life Technologies) for 21 day. Each subgroup was made in three replicates. Extracellular matrix formation in pellets was assessed by histological methods.

### Histology of Chondrogenic Differentiation in Cell Pellets

For histochemical and immunohistochemical analysis, chondrogenic differentiation pellets were fixed in 10% neutral buffered formalin and embedded into paraffin. Histological sections of 3-μm thickness were deparaffinised and processed for standard staining with safranin-O (Sigma-Aldrich). Immunohistochemical staining with antibodies against collagen type II (Abcam) was performed after antigen retrieval with citrate buffer pH 6.0 at +98°C for 20 min and endogenous peroxidase blocking with 0.3% hydrogen peroxide for 15 min at room temperature. ABC staining kit (Santa Cruz) and 3.3-diaminobenzidine as a chromogen were used. Stained sections were evaluated and blindly scored and independently by two histology experts.

### Statistical Analysis

Statistical difference between groups was evaluated using one-way analysis of variance (ANOVA) and *post-hoc* Tukey-Kramer Multiple-Comparison Test and the Student's *t*-test using NCSS software. *p* ≤ 0.05 was considered as statistically significant.

## Results

### Proliferation of Chondrocytes and BMMSCs

**Figure 1:**
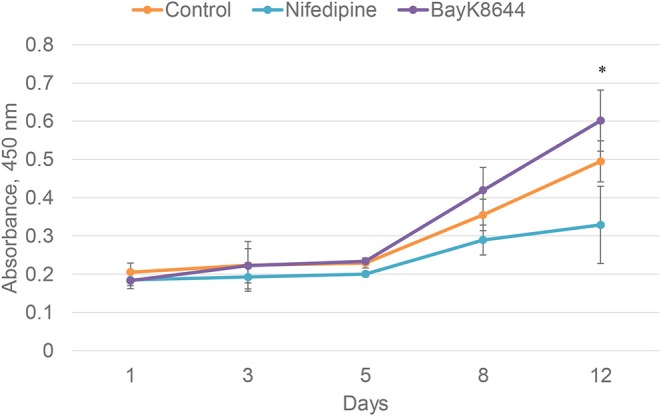
Proliferation of chondrocytes after the treatment with nifedipine (10 μM) and BayK8644 (10 μM) for 1, 3, 5, 8, and 12 days. CCK-8 viability and cytotoxicity assay. The absorbance of reduced formazan dye, produced by living cells, is presented. Absorbance measured at 450 nm. *p < 0.05.

**Figure 2:**
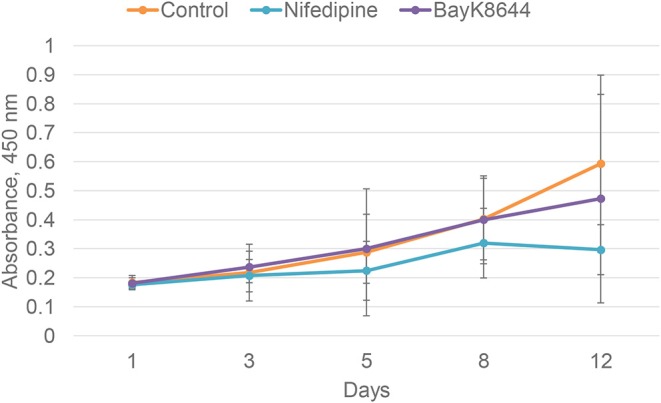
Proliferation of BMMSCs after the treatment with nifedipine (10 μM) and BayK8644 (10 μM) for 1, 3, 5, 8, and 12 days. CCK-8 viability and cytotoxicity assay. The absorbance of reduced formazan dye, produced by living cells, is presented. Absorbance measured at 450 nm.

To evaluate the effects of nifedipine and BayK8644 on proliferation of chondrocytes and BMMSCs, these compounds were added to cell culture medium and cell proliferation was measured during 1, 3, 5, 8, and 12 days. Nifedipine significantly decreased proliferation of chondrocytes only during 12th day of cultivation while BayK8644 did not have any significant effect (Figure 1). VOCC regulators had no significant effect on BMMSC's proliferation (Figure 2).

### Alterations in Cellular Metabolism as a Response to Nifedipine and BayK8644

**Figure 3:**
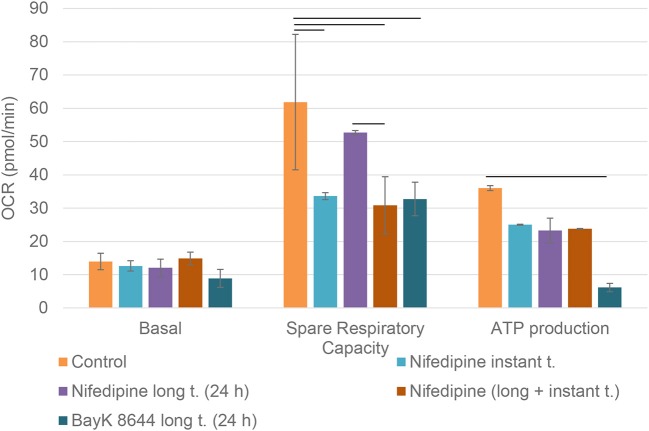
Mitochondrial respiration capacity in chondrocytes. The cells were treated with nifedipine (10 μM) and BayK8644 (10 μM) for 24 h (long t.), and additionally treated with nifedipine (10 μM) during the measurement (instant t.). Basal, spare respiratory capacity and ATP production are presented. OCR, oxygen consumption rate. Horizontal bars represent *p* < 0.05.

Mitochondrial spare respiratory capacity in chondrocytes was significantly reduced by an instant treatment with nifedipine during measurement, but not by long treatment. However, ATP production was significantly downregulated by all of the treatments, especially BayK8644. Moreover, BayK8644 also significantly reduced spare respiratory capacity in chondrocytes (Figure 3).

**Figure 4:**
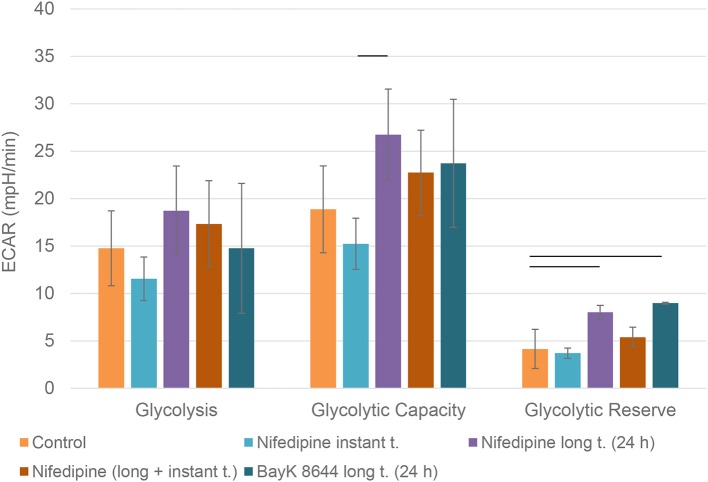
Glycolysis in chondrocytes. The cells were treated with nifedipine (10 μM) and BayK8644 (10 μM) for 24 h period (long t.), and additionally treated with nifedipine (10 μM) during the measurement (instant t.). ECAR, extracellular acidification rate. Horizontal bars represent *p* < 0.05.

Nifedipine long treatment significantly increased glycolysis in chondrocytes (Figure 4). Furthermore, nifedipine and BayK8644 significantly increased glycolytic reserve, which is an important parameter in cells energy metabolism, as it indicates the capability of a cell to respond to an energetic demand as well as shows how close the glycolytic function is to the cell's theoretical maximum.

**Figure 5:**
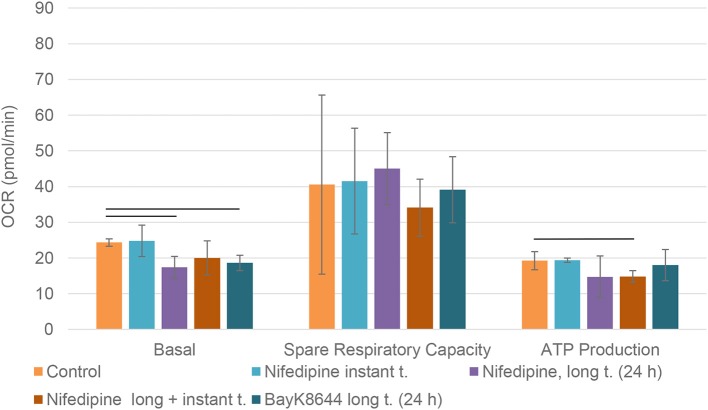
Mitochondrial respiration in BMMSCs. The cells were treated with nifedipine (10 μM) and BayK8644 (10 μM) for 24 h period (long t.), and additionally treated with nifedipine (10 μM) during the measurement (instant t.). OCR, oxygen consumption rate. Horizontal bars represent *p* < 0.05.

Long term (24 h) incubation with nifedipine downregulated basal mitochondrial respiration in BMMSCs, while instant treatment had no significant effect (Figure 5). Pre-treatment with BayK8644 also downregulated basal respiration. Nifedipine resulted in a repression of ATP production, but only a combination of long and instant treatments reached statistical significance. None of the treatments had any significant effects on spare respiratory capacity.

**Figure 6:**
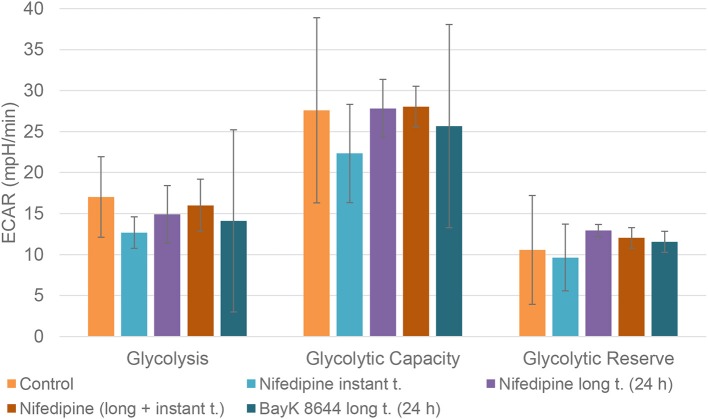
Glycolysis in BMMSCs. The cells were treated with nifedipine (10 μM) and BayK8644 (10 μM) for 24 h period (long t.), and additionally treated with nifedipine (10 μM) during the measurement (instant t.). ECAR, extracellular acidification rate.

Neither nifedipine, nor BayK8644 had a significant effect on glycolytic capacity or glycolytic reserve in BMMSCs (Figure 6).

### Transmission Electron Microscopy Study of Cartilage Explants Treated With Nifedipine

**Figure 7:**
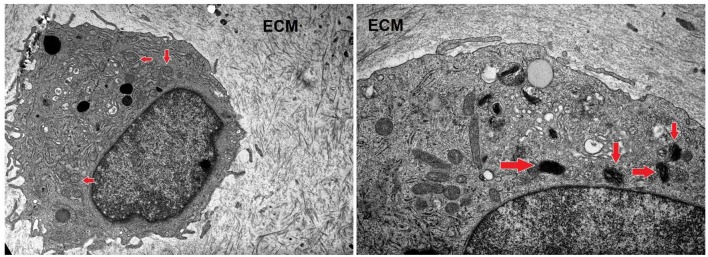
Sections of cartilage explants derived from cartilage samples, untreated (control), and treated with nifedipine (10 μM). Control sample –unchanged chondrocyte in ECM, with normal, light mitochondria (marked by arrows). Magnification × 4 k. nifedipine-treated sample—matrix of some mitochondria is darker (marked by arrows). Magnification × 10 k. ECM, extracellular matrix.

After a long-term incubation with nifedipine (7 days), cartilage explants were analyzed by transmission electron microscopy, as shown in Figure 7. In controls there were few or no electron-dense mitochondria. In nifedipine-treated samples some mitochondria became electron-dense, clusters of contiguous mitochondria (left side of micrograph) remain normal. Augmented electron-density of mitochondrial matrix might reflect the dropped activity of mitochondrias.

### Nifedipine Treatment Increased Intracellular Calcium Levels in Chondrocytes and BMMSCs

**Figure 8:**
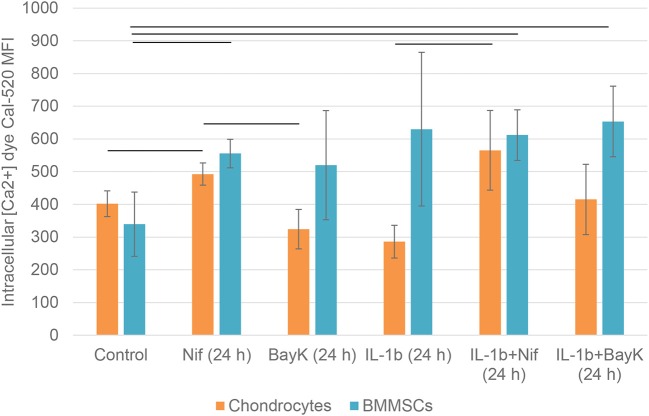
Mean fluorescence intensity (MFI) of intracellular calcium Ca^2+^ dye Cal-520 in chondrocytes and BMMSCs after treatment with nifedipine (10 μM) (Nif), BayK8644 (10 μM) (BayK), IL-1β (1 ng/mL) (IL-1b), IL-1β (1 ng/mL) + BayK8644 (10 μM) (BayK), and IL-1β (1 ng/mL) + nifedipine (10 μM) (Nif) for 24 h. Horizontal bars represent *p* < 0.05.

To investigate the effects of nifedipine and BayK8644 on their direct targets—VOCC, changes in intracellular calcium concentrations were studied. The cells were additionally incubated with IL-1β, which is an important cytokine in the early stages of OA, as well as IL-1β + nifedipine and IL-1β + BayK8644. As shown in Figure 8, nifedipine significantly increased intracellular calcium concentration Ca^2+^ in both cell types. While IL-1β significantly decreased intracellular Ca^2+^ in chondrocytes, IL-1β + nifedipine treatment increased it as compared to control. BayK8644 treatment didn't significantly change Ca^2+^ in chondrocytes, as compared to control. In BMMSCs, nifedipine, IL-1β + nifedipine, and IL-1β + BayK8644 significantly increased it.

### Nifedipine Increased Nitric Oxide Accumulation in Chondrocytes and BMMSCs

**Figure 9:**
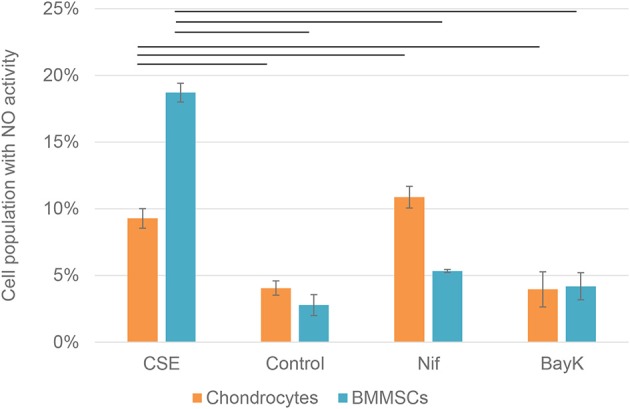
Nitric oxide (NO) activity in chondrocytes and BMMSCs. Treatment with cigarette smoke extract (CSE) (1 μg/mL), DMSO (Control), nifedipine (10 μM) (Nif), BayK8644 (10 μM) (BayK) for 24 h. Horizontal bars represent *p* < 0.05.

**Figure 10:**
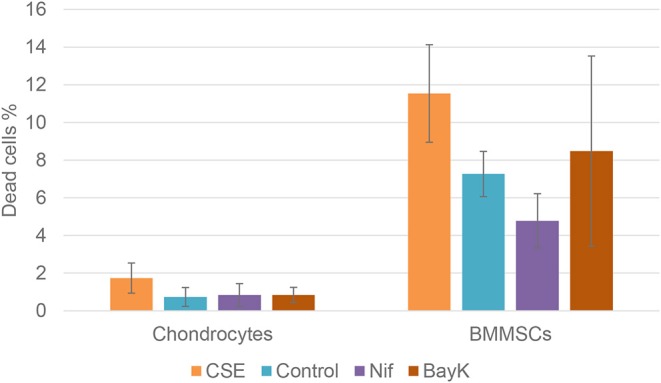
Chondrocyte and BMMSC dead cell population after treatment with cigarette smoke extract (CSE) (1 μg/mL), nifedipine (10 μM) (Nif), BayK8644 (10 μM) (BayK) for 24 h; 7-AAD (flow cytometry).

Nitric oxide (NO) activity in chondrocytes and BMMSCs was analyzed due to its direct roles in mitochondria functions and inflammation processes. NO activity increased when cigarette smoke extract (CSE) was added. CSE was chosen as positive control since it has been known to induce NO in mesenchymal stem cells (MSCs) from previous experiments (unpublished data). Nifedipine significantly increased NO activity in chondrocytes and BMMSCs (Figure 9), as compared to control. BayK8644 had no significant effect on both cell types. Furthermore, the viability of both cell types was within the normal range, as determined by the levels of dead cells by flow cytometry (Figure 10). Noteworthy, it was not increased after the cell incubation with nifedipine or BayK8644, as compared to the respective unstimulated controls.

### Chondrogenic Differentiation of Nifedipine and BayK8644 Treated Chondrocytes and BMMSCs

**Figure 11:**
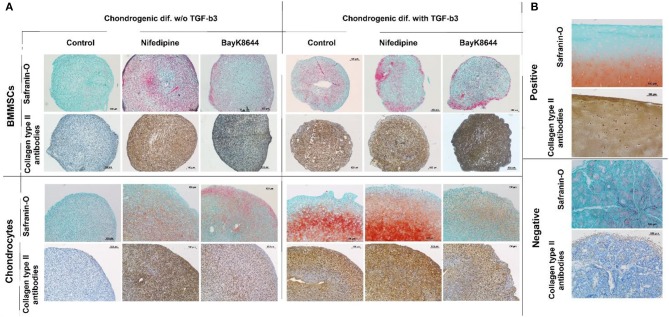
Chondrogenic differentiation of BMMSCs and chondrocytes. Cells were incubated in chondrogenic differentiation medium with and without TGFβ3. Each group was divided into: Control, treated with nifedipine (10 μM) and treated with BayK8644 (10 μM). **(A)** Histological sections of cell pellets, stained with Safranin-O and anti-collagen type II antibodies, ×100 magnification. **(B)** Control staining for histological sections with Safranin-O and anti-collagen type II antibodies, where positive is cartilage tissue and negative—salivary gland.

Treatment with nifedipine stimulated chondrogenic differentiation and ECM production both in chondrocytes and in BMMSCs in the absence of TGFβ3, as demonstrated by deposition of both glycosaminoglycans (GAGs) and Collagen type II, stained with Safranin-O and anti-collagen type II antibodies, respectively (Figure 11). In the presence of TGFβ3, nifedipine upregulated production of GAGs and collagen type II only in chondrocytes, whereas on BMMSCs had no effects. Noteworthy, macroscopically, pellet sizes of nifedipine treated samples were larger in chondrocytes and BMMSCs, as compared to respective controls, while no essential size differences were observed in all samples cultured with TGFβ3.

In the absence of TGFβ3, BayK8644 also stimulated cartilage ECM production in both chondrocytes and BMMSCs, however the effects were much weaker, as compared to those of nifedipine (Figure 11). In the presence of TGFβ3, the amount of collagen type II in BMMSC-derived pellets seemed to be upregulated, whereas, in chondrocytes, in contrast, BayK8644 even inhibited GAGs and collagen type II production.

## Discussion

In the present study, we were seeking to elucidate the role of antihypertensive drug nifedipine on functions and energy metabolism in chondrocytes and BMMSCs. Nifedipine is a VOCC antagonist, therefore, in order to understand if those effects were mediated through Ca^2+^ channels, we have also compared its effects to those of agonist BayK8644. Nifedipine and BayK8644 were used at a most efficient standard concentration of 10 μM in all experiments, as previously described (19–23).

The downregulation of proliferation was observed in both chondrocytes and BMMSCs, however only in chondrocytes it was significant. This may signify potential cytotoxic or cytostatic effects of Nifepidine. It has also been shown that nifedipine inhibited rat arterial smooth muscle cell proliferation *in vitro* (24). On the other hand, chondrogenic differentiation is also associated with cell cycle arrest (25), suggesting that the reduction of proliferation by nifedipine might signify a switch toward chondrogenesis and initiation of ECM production in both cell types. Moreover, cytotoxic effects of nifedipine or BayK8644 were not observed, as demonstrated by unaltered low levels of dead cells, using 7-AAD staining.

VOCC agonist BayK8644 had no inhibitory effect on cell proliferation and even tended to stimulate it in chondrocytes. However, these results are in stark to the published data on gingival fibroblasts which showed a better proliferation rate when treated with nifedipine, as compared to the untreated controls (26, 27). Similarly, nifedipine promoted cell proliferation in breast cancer cell lines (28, 29).

In response to nifedipine and BayK8644, changes in cell metabolism were analyzed, particularly mitochondrial respiration and glycolysis, that are the main energy generating processes in cells.

The main goal to analyze both, long and instant application of nifedipine was the lack of data on the duration of effects of nifedipine. We wondered if stimulation by nifedipine could affect metabolism for many hours or even several days or whether the effects are more temporal. In chondrocytes, the application of nifedipine for either instant or long (24 h) duration significantly downregulated ATP production, suggesting blockage of mitochondrial respiration. Noteworthy, both spare respiratory capacity and glycolytic capacity were significantly lower after instant nifedipine treatment, as compared to the 24 h application suggesting that those parameters respond immediately and then gradually are compensated. Conversely, only long nifedipine treatment augmented glycolytic reserve, suggesting an efficient switch to compensatory energetic production in chondrocytes.

BMMSCs responded differently: only long (24 h) application downregulated basal respiration level and ATP production, whereas no induction of glycolysis was observed.

Altogether these data suggest that nifedipine may lead to an energetic arrest in BMMSCs and chondrocytes, which could also, at least in part, account for the reduced proliferation, as was shown in the study with berberine in HepG2, HeLa, and Hepa1-6 cell lines (30). In agreement to that, the analysis of chondrocyte mitochondria by electron microscopy in cartilage explant histological sections has also suggested that part of mitochondria lose their activity in response to nifedipine.

Unexpectedly, the VOCC agonist BayK8644 had similar metabolic effects to nifedipine, including induction of glycolytic reserve in chondrocytes and blockage of ATP production in both chondrocytes and BMMSC. These data imply that additional mechanisms than modulation of Ca^2+^ channels may be involved in the effects of antihypertensive drug nifedipine on BMMSCs and chondrocytes.

Therefore, the roles of nifedipine and BayK8644 to their direct target changes in intracellular Ca^2+^ concentration were analyzed by flow cytometry. To represent the osteoarthritic environment, effects of IL-1β, one of the crucial pro-inflammatory factors during OA (31) on Ca^2+^ concentration were also investigated.

Intracellular calcium levels were not decreased, but unexpectedly increased in nifedipine, while not BayK8644 treated cells of both types. These data are in agreement to the previously observed upregulation of intracellular calcium by nifedipine from ryanodine receptor-mediated endoplasmic reticulum stores of neonatal neuromuscular junction in rats, suggesting a compensatory mechanism in cells (32). Increase in cytoplasmic calcium has been also reported in pulmonary arterial smooth muscle cells stimulated with the dihydropyridine Ca^2+^ channel blockers (33). Noteworthy, nifedipine was shown to increase cytoplasmic calcium by stimulating the activity of Ca^2+^-sensing receptor in those cells, independently of their blocking (or activating) effect on calcium channels (33). Furthermore, similar increase in intracellular calcium was also determined in porcine aortic endothelial cells that do not express L-type calcium channels (34), suggesting potential involvement of additional mechanisms of nifedipine action in different cell types.

To further understand the mechanisms by which nifedipine blocks mitochondrial function, the production of NO was investigated. Nifedipine has been shown to increase endothelial NO bioavailability (13), and upregulating intracellular calcium in striatal neurons (35), whereas inhibition of mitochondrial activity by NO has been demonstrated (36). Similarly, in the present study, NO activity was stimulated by nifedipine in BMMSCs and particularly chondrocytes, suggesting that NO at least in part may account for the effects of nifedipine on metabolism in both tested cell types. Conversely, BayK8644 had no effect on NO activity, although it was the most potent blocker of ATP in chondrocytes, suggesting that different mechanisms might be implicated in its action on mitochondrial respiration.

Finally, the effects of nifedipine and BayK8644 on chondrogenesis and extracellular matrix production were assessed in chondrocytes and BMMSCs. Stimulation with either nifedipine or BayK8644 resulted in induction of collagen type II and proteoglycan production even in the absence of TGFβ3, although the effects of VOCC inhibitor BayK8466 were less pronounced. Noteworthy, there were no signs of cell viability reduction in nifedipine or BayK8644 treated groups, as an extracellular matrix in cell pellets was well-developed and evenly organized, indicating that the quantity and functional efficiency of the cells producing it was not reduced.

To the best of our knowledge, we are the first to study the effects of Ca^2+^ channel modulators on the metabolic activity of chondrocytes and BMMSCs, and our novel observations may reveal novel and promising targets for the treatment of OA. Nevertheless, a deeper analysis of the effects of VOCC inhibitors on chondrocyte hypertrophy, inflammatory activity, and modulation of catabolic enzymes involved in cartilage degradation, including matrix metalloproteinases and ADAMTS (a disintegrin and metalloproteinase with thrombospondin motifs) may help us elucidate how Ca^2+^ channel inhibition may be implicated in cellular dysfunction and OA pathogenesis.

## Conclusion

Taken together, we conclude that the antihypertensive drug nifedipine inhibits mitochondrial respiration in both chondrocytes and BMMSCs, and that these effects may be associated with the increased NO production and pro-inflammatory activity. Glycolytic capacity was enhanced only in chondrocytes, suggesting that these cells have the capacity to switch from oxidative phosphorylation to glycolysis and alter their metabolic activity in response to VOCC inhibition. Finally, nifedipine had positive effects on the production of collagen type II and proteoglycans in both cell types, implying potentially beneficial anabolic responses in articular cartilage. These results highlight a potential link between antihypertensive drugs and cellular changes that occur in chondrocytes in OA cartilage.

## Data Availability Statement

The data that support the findings of this study are available from the corresponding author, EB, upon reasonable request.

## Ethics Statement

The studies involving human participants were reviewed and approved by Vilnius Regional Committee on Biomedical Research Ethics. The patients/participants provided their written informed consent to participate in this study.

## Author Contributions

IU, EBe, GR, EBa, and JD: writing-original draft preparation. GK and NP: patient selection, tissue sample preparation, and manuscript editing. EBe: study design and supervision. AM: conceptualization, supervision of metabolic studies, and manuscript editing. ZM: transmission electron microscopy study, histological analysis of chondrogenic differentiation pellet samples.

### Conflict of Interest

The authors declare that the research was conducted in the absence of any commercial or financial relationships that could be construed as a potential conflict of interest.

## Abbreviations

1.4-DHP: 1.4-Dihydropyridines
2-DG: 2-deoxy-glucose
7-AAD: 7-Aminoactinomycin D
ADAMTS: A disintegrin and metalloproteinase with thrombospondin motifs
BMMSCs: bone marrow mesenchymal stem cells
iCa^2+^: intracellular calcium
CCK-8: cell counting kit-8
CSE: cigarette smoke extract
CVD: cardiovascular disease
DMEM: Dulbecco's modified Eagle's medium
DMSO: dimethyl sulfoxide
ECAR: extracellular acidification rate
ECM: extracellular matrix
FBS: fetal bovine serum
FCCP: carbonyl cyanide p trifluoromethoxyphenylhydrazone
FGF-2: fibroblast growth factor 2
IL-1β: interleukin 1β
MSCs: mesenchymal stem cells
NO: nitric oxide
OA: osteoarthritis
OCR: oxygen consumption rate
PBS: phosphate buffer saline
ROS: reactive oxygen species
TGF-β: transforming growth factor β
VOCCs: voltage-operated calcium channels.
